# A Comparison of the Dimensional Characteristics and Plasma Parameters of Different Centrifuges Used for the Preparation of Autologous Platelet Concentrates: A Randomized Correlational Study

**DOI:** 10.3390/ma18020414

**Published:** 2025-01-17

**Authors:** Leandro Lécio de Lima Sousa, Daiana Fermiano Brunale, Gustavo Vicentis Oliveira Fernandes, Gabriela Giro, Marcelo Faveri

**Affiliations:** 1 Department of Periodontology, Dental Research Division, Guarulhos University, Guarulhos 07023-070, Brazil; 2 Periodontics, A.T. Still University-Missouri School of Dentistry & Oral Health, St. Louis, MO 63104, USA

**Keywords:** dental implants, platelet-rich fibrin, platelet concentrates, centrifuge, blood serum values

## Abstract

Objective: The objective of this study was to evaluate autologous platelet-rich fibrin (PRF) membrane weights and measurements after production by different centrifuges. Moreover, the values obtained with blood cellular components were correlated. Methods: Twelve systemically healthy participants underwent dental implant surgery associated with PRF membranes as the graft biomaterial at the implant site. Prior to the surgical procedure, the chosen participants underwent blood count and coagulogram tests and presented on the surgical day. Nine tubes containing 10 mL of venous blood were collected from each individual. The tubes were randomly distributed and positioned in three different centrifuges: (C1) the Intra-lock L-PRF Process, (C2) the Kasvi Digital, and (C3) the PRF Montserrat. PRF membrane processing was carried out as described by each manufacturer. After the processing steps, the prepared wet PRFs (initial) were placed in the container (box) designated by the manufacturer for the obtention of PRF membranes. The weights and measurements of the “wet” PRFs (initial) and membranes (final) were obtained using a precision scale and digital caliper in an aseptic environment. The data were compared, and the statistical differences were analyzed using the Friedman test and the Dunn post hoc test; Pearson correlation tests were performed between macroscopic data and data from serum tests; statistical significance was set at 5% (*p* < 0.05). Results: 108 blood collection tubes were collected. The average harvest time for each tube individually was 21.5 ± 9.9 s. The average time for blood collection (nine tubes) from each of the 12 individuals was 193.1 ± 72.4 s (*p* = 0.728). The average values were very similar between the centrifuges, both for the measurements and weights of the “plugs” as well as for the linear measurements (*p* > 0.05). Regarding the wet weights and the linear averages of the PRF membranes, it was observed that the wet PRF weights varied from 0.22 to 0.25 mg and the linear measurements from 24.1 to 26.7 mm, with no statistical differences between centrifuges (*p* > 0.05). The data presented by centrifuges C1 and C2 were more homogeneous, delivering a value of less than 25% variability compared to the C3 centrifuge, which achieved values greater than 33%. Conclusions: The proposed macroscopic dimensional evaluation found no differences between the autologous platelet concentrates obtained by different centrifuges, and no correlation was found between these PRFs and the patients’ blood counts.

## 1. Introduction

The expression “tissue engineering” emerged from the use of devices to facilitate tissue regeneration, combining three key elements: matrixes (scaffolds—three-dimensional [3D] structures that serve to support cell growth), signaling molecules (such as growth factors), and cells (e.g., osteoblasts, fibroblasts, or other populations suitable for the regeneration of injured tissue) [1,2]. All of these work together synergistically during the repair and/or regeneration process. Then, the trend is that many products attempt to adapt to these natural mechanisms to improve tissue response/healing [3].

Platelets represent a fragment of megakaryocytes, constituting elements circulating in the bloodstream. A human adult has nearly one trillion circulating platelets, with average lifespans of 8 to 10 days. These circulating elements play important key roles: responding to vessel injuries, regulating angiogenesis, and innate immunity. In addition, platelet derivates, autologous platelet concentrates (APCs), have become an option for clinical applications in various areas such as guided periodontal procedures and bone regenerations, sinus floor elevation, and support for regeneration of the pulp–dentine complex [4]. They are autologous preparations [5,6] that eliminate the risk of disease transmission and immunological reactions without the risk of infection, benefiting the patient [7].

During this period, platelets secrete granules that contain proteins, promoting blood clotting through factors such as β-thromboglobulin, fibronectin, thrombospondin, fibrinogen, growth factors, fibrinolysis inhibitors, and immunoglobulins [8]. During this process, cytokines are released that stimulate cell migration and proliferation within the fibrin network. Growth factors, such as platelet-derived growth factor (PDGF) and transforming growth factor-beta (TGF-β), are also released, regulating the inflammatory and fibrous healing process, and are responsible for the migration, proliferation, and survival of the mesenchymal cells that are accountable for the healing tissue remodeling mechanism [9].

Del Corso et al. [10] and Ehrenfest et al. [11] classified APCs into four families: P-PRP (pure platelet-rich plasma), L-PRP (platelet- and leukocyte-rich plasma), P-PRF (pure platelet-rich fibrin), and L-PRF (leucocyte- and platelet-rich fibrin). The first two are platelet suspensions, respectively, with or without leukocytes that can be used in liquid or gel form after activation using thrombin and calcium chloride (anticoagulant), centrifuged in two steps, resulting in a not very well-structured fibrin network. PRF is the development of the simplified thrombin-free technique [12], obtained with one centrifugation step. Ehrenfest et al. [13] stated that PRF is a dense autologous fibrin biomaterial in solid form loaded with autologous cells, a highly biocompatible and inductive 3D fibrin network for tissue engineering applications [14]. It is an optimized natural clot [15] after blood clotting in a homogeneous and strong fibrin matrix without red blood cells but with circulating platelets, leukocytes, and undifferentiated mesenchymal cells. It produces an improvement over the natural blood clot, stimulating healing potential and promoting the regeneration of soft and bone tissues [10].

Platelet-rich fibrin (PRF), the second APC generation, is obtained through a natural process, unlike PRP, due to not being associated with any chemical reaction [9,16]. PRF is highly biodegradable [14]. PRF membranes have been placed subcutaneously in mice and, within two weeks, were replaced by dense collagen. The use of PRF in dental sockets after extraction has demonstrated a better healing response for bone and soft tissues [12]. In vivo studies have shown the use of PRF combined with other biomaterials (bioglass, hydroxyapatite [HA], and β-tricalcium phosphate [β-TCP]) presenting significant new bone gain [17]. Anwandter et al. [18] observed that PRF has shown similar results for bone crest preservation procedures obtained with heterogeneous or allogeneic grafts and is superior even to alloplastic grafts or natural healing and that it can be effective at the same level as bone substitutes, but without leaving behind graft particles and without a high cost. Moreover, platelet concentrates provide additional benefits during post-surgical and second-intention wound healing [19].

Castro et al. [20], in a systematic review, extensively analyzed the regenerative potential of PRF during periodontal surgery; their meta-analysis showed significant clinical benefits for the use of PRF for the treatment of intraosseous defects and furcation defects and similar results when PRF membranes replaced a connective tissue graft (CTG) during periodontal plastic surgery. In an umbrella review, Fernandes et al. [21] confirmed that, even though APC (the one-centrifugation method) exhibited no superior results compared to CTG, it is a promising substitute for CTG in root coverage procedures. Moreover, Otero et al. [22] showed that the use of PRF in sinus augmentation had favorable outcomes, demonstrating the amplification of the use of PRF. In another study [23], the authors conducted a systematic review to analyze the effect of PRF on bone regeneration and osseointegration. Studies have shown around a 20% increase in bone regeneration when using PRF compared to controls, highlighting its potential benefit in osseointegration. Otherwise, Fernandes et al. [24], in a split-mouth randomized controlled study, did not find significant results using PRF liquid, which had similar results to the control group.

Many types and variations of the original PRF have emerged, presenting an interesting evolution [25,26]. Ehrenfest et al. [27] analyzed the differences between the different protocols for obtaining PRF and the original L-PRF using different centrifuges. The authors observed distinct biological components, demonstrating a clear difference between the platelet aggregates obtained, mainly in relation to the structural part of the fibrin. The authors described that this factor may influence the release threshold of growth factors. Furthermore, researchers have observed that the choice of equipment could affect the PRF’s weight and size and possibly impact the final cellular content and biological effects [28].

Thus, the objective of this study was to evaluate autologous platelet-rich fibrin (PRF) membranes’ weights (mg) and measurements (mm) after production by different centrifuges, correlate the values with blood cellular components, and determine whether the type of centrifuge influences PRF production.

## 2. Material and Methods

### 2.1. Study Design

This study followed the Declaration of Helsinki (1975, updated 2013), the standards of the Brazilian National Health Council (Resolution no. 466/12), and was approved by the local Ethics Committee (UNG University; number: 56949216.8.0000.5506). Participants (who needed oral rehabilitation in the posterior region of the mandible) were informed of the objectives of the study, its risks, and benefits, including the types of clinical measurements and blood sample collection. Those who agreed to participate in the study and signed the Informed Consent form after anamnesis and case management were included and received the proposed surgical therapy.

### 2.2. Eligibility Criteria

The inclusion criteria were: (1) presenting the absence of at least one posterior dental element of the mandible (premolars and/or molars); (2) presenting the need for rehabilitation with at least one implant in the posterior region of the mandible, with bone availability for the insertion of dental implants; (3) not presenting periodontal disease at the time of implant surgery; and (4) not presenting any systemic disease that contraindicates implant insertion surgery. The patients who met the criteria below were disregarded as a study sample: (1) presenting a history of debilitating chronic diseases, such as hepatitis, rheumatic fever, diabetes, immune or blood disorders, or other diseases that contraindicate surgery; (2) autoimmune diseases with oral manifestations; (3) presenting a history of osteometabolic disorders, except osteoporosis/osteopenia, which would interfere with the healing of dental implants; (4) the need for regenerative surgeries prior to implant insertion; (5) having areas that have received bone grafts; (6) the presence of fresh alveoli at the implant placement site; (7) the use of steroidal anti-inflammatory drugs in the three months before the study; and (8) smokers.

### 2.3. Experimental Design

In the experimental design, at the beginning, all individuals underwent anamnesis, a clinical diagnostic examination (screening), and a tomographic examination. At this time, clinical data on the teeth present in the oral cavity were obtained, and an impression was taken to create a surgical guide. Patients were asked for blood count and coagulogram tests carried out at the Carlos Chagas Laboratory (Guarulhos, SP, Brazil) prior to the surgical procedure (1 day before surgery). All necessary biosafety protocols for the procedure were followed to install the selected implants. The planned implants were installed according to the manufacturer’s instructions, and two-stage surgical implants were used. All bone drilling was performed under abundant irrigation with a sterile saline solution. After the insertion of the implants, the PRF membrane, previously collected, prepared, and measured, was cut according to the number of implants to be covered and positioned on the bone crest in the region of the installed implant for the subsequent coaptation of the edges of the flap using sutures (5/0 nylon threads) in simple interrupted stitches.

### 2.4. Blood Collection

Venous blood was collected from the participants and taken from the forearm using a 21 G scalp for vacuum blood collection (21Gx3/4x7, BD, Franklin Lakes, NJ, USA). For collection, 10 mL tubes (BD Vacutainer Serum, BD, Franklin Lakes, NJ, USA) were used without the addition of anticoagulants, bovine thrombin, or any other chemical agent (natural blood clot formation). The harvest was always carried out in the morning, between 8 and 10 a.m., and the procedures were always performed by only one professional trained in venous blood collection to standardize procedures.

On this occasion, nine tubes containing approximately 10 mL of venous blood were collected from each individual. Collections were sequenced every three tubes (blocks of 3 tubes harvested) and immediately allocated to the respective centrifuges. The order of the collection blocks and the centrifuges used were randomized using a table generated by statistical software (SPSS version 23.0). The venous collection procedures were timed to evaluate the influence of the collection time on the processing of the membranes. After collection (of a block of 3 tubes), in a maximum time of 2 min, the collected tubes were added to the centrifuges, and a fourth tube containing 10 mL of water was added to each centrifuge to balance the centrifugation procedure. The centrifuges used were: Test 1 (C1)—the L-PRF Intra-lock Process (Grevenbroich, Germany); Test 2 (C2)—the Kasvi Digital (Beijing, China); and Test 3 (C3)—the PRF Montserrat (Shanghai, China).

### 2.5. PRF Protocol Used

Each centrifuge received three venous collection tubes and a tube containing 10 mL of water. The centrifugation protocols for each centrifuge are described below and were carried out in accordance with the manufacturers’ recommendations: Test 1 (C1)—the Intra-lock L-PRF Process—a centrifugation process of 12 min at 2700 rpm; Test 2 (C2)—the Kasvi Digital—a centrifugation process of 10 min at 2500 rpm; Test 3 (C3)—the PRF Montserrat—a centrifugation process of 10 min at 2500 rpm.

After removing the PRFs from each centrifuge, they were removed from the tube and placed directly on a solid surface and immediately measured according to their linear extension (length) using a digital caliper (ABS Absolute Digital Caliper 6 In.—MITUTOYO-500-171-30B, Tokyo, Japan); subsequently, the PRFs had their “wet weight” (of the raw product obtained) measured on a precision scale (9094 Plus Toledo do Brasil, São Bernardo do Campo, SP, Brazil)—the PRF was gently transferred from the solid surface to the scale. Then, the PRFs were gently transferred and placed in a box suitable for forming autologous membranes (Supremo Odonto, Carapicuiba, SP, Brazil). Afterward, the same linear measurement (length) and “wet weight” procedures were conducted on the membranes formed.

### 2.6. Statistical Analysis

The mean and standard deviation (SD) of the macroscopic measurements (mg and mm) and venous blood collection times were computed for each individual and each sample from the different centrifuges used. Statistical differences were analyzed using the Friedman test and Dunn post hoc test. Pearson correlation tests were performed between macroscopic data and data from serum tests conducted on the individuals. Statistical significance was set at 5% (*p* < 0.05).

## 3. Results

**Figure 1:**
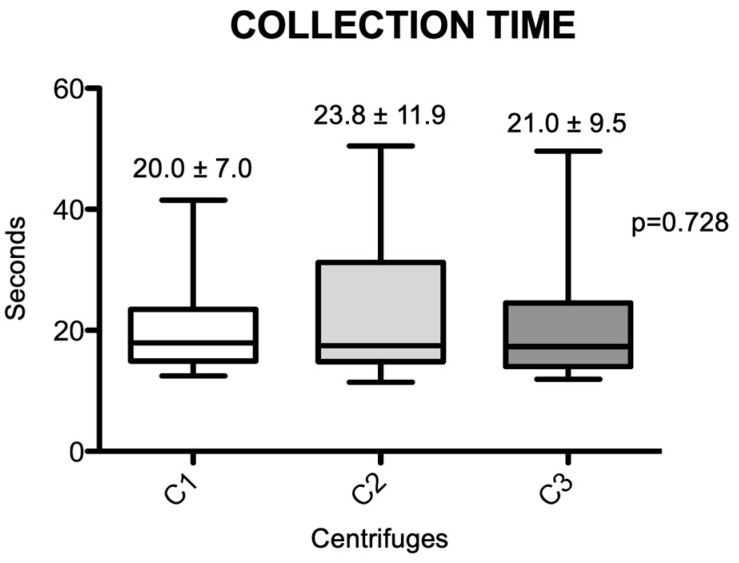
Box-plots of the differences in venous blood collection times achieved for the different centrifuges. Significant differences between the groups were measured using the Friedmann test (*p* = 0.728). (C1): the L-PRF Intra-lock Process, Germany; (C2): the Kasvi Digital, China; (C3): the PRF Montserrat, China.

In total, 50 individuals were initially selected; subsequently, after deep evaluation, 12 (6 females and 6 males) were selected to participate in the present study, with an average age of 36.1 years. A total of 108 blood collection tubes were collected, and the average collection time for these tubes was 21.5 ± 9.9 s. The average time for blood collection (nine tubes) from each of the 12 individuals was 193.1 ± 72.4 s. Figure 1 shows the average collection time for each randomized tube in the three centrifuges tested. It was observed that there was no difference in the average tube collection time between the centrifuges (*p* = 0.728), with group C1 having a lower average (20.0 ± 7.0 s), followed by group C3 (21. 0 ± 9.5 s) and C2 (23.8 ± 11.9 s), but without statistical differences (*p* = 0.728).

**Figure 2:**
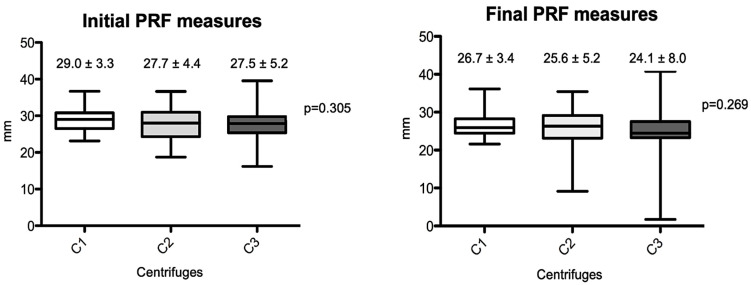
Box-plots of the differences between PRF length measurements produced by the different centrifuges (initial and final results). (C1): the L-PRF Intra-lock process, Germany; (C2): the Kasvi Digital, China; (C3): the PRF Montserrat, China.

**Figure 3:**
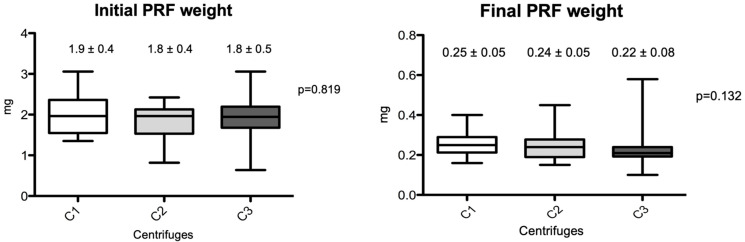
Box-plots of the differences between PRF weights produced by the different centrifuges (initial and final results). (C1): the L-PRF Intra-lock process, Germany; (C2): the Kasvi Digital, China; (C3): the PRF Montserrat, China.

The macroscopic measurements are shown in Figure 2 and Figure 3. Linear measurements (lengths) were made from the PRFs obtained by the three centrifuges tested, as well as their wet weights, before and after membrane processing. Figure 2 shows the initial and final average measurement distribution values by centrifuge, and Figure 3 shows the initial and final average weights. No statistical differences were observed among the centrifuge groups for any of the parameters analyzed (*p* > 0.05). The final results showed practically the same weights and linear measurements in the intragroup analysis after the preparation of the PRF membranes; the average values were very similar in relation to the PRF wet weights and the linear averages (intergroup, initial evaluation), whereas the PRF membranes already prepared had a non-significant average weight variance from 0.22 to 0.25 mg (*p* = 0.132) and linear measurements ranged from 24.1 to 26.7 mm (*p* = 0.269).

**Figure 4:**
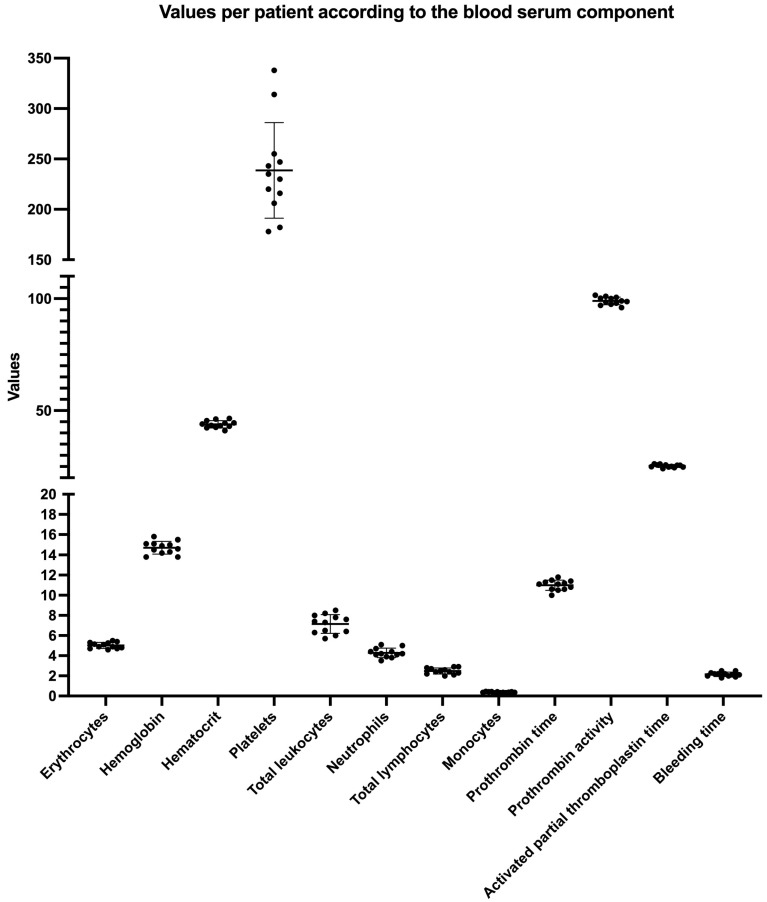
A graphical presentation of the values obtained per patient according to the observed blood serum component. The data are presented per patient with the mean and standard deviation (SD).

The coefficient of variability among centrifuges for the four macroscopic measurements carried out in the present study is presented in Table 1. It is possible to observe that the C1 and C2 exhibited more homogeneous data than C3. The lowest dispersion values were observed for C1, and the most heterogeneous data were found for C3. Table 2 presents the average serum values of the blood components of the individuals enrolled (Figure 4). No significant correlations were found between the weight and length measurements for the PRF wet (initial), membrane (final), and the patient’s blood component data independently of the centrifuge used.

## 4. Discussion

PRF is an autologous platelet concentrate obtained through a simple centrifugation process, which allows for the concentration of platelets and growth factors without using anticoagulants or additives [29]. Variants of PRF, such as injectable platelet-rich fibrin (i-PRF) and advanced platelet-rich fibrin (A-PRF), have been developed to enhance its clinical applications [25,26]. For instance, L-PRF, which contains a higher concentration of leukocytes, has already demonstrated improvements in wound healing and tissue regeneration due to its cytokine-rich profile [30]. The versatility of PRF and its variants has led to their application in various dental procedures, including alveolar ridge preservation, sinus augmentation, and periodontal regeneration, demonstrating significant improvements in healing outcomes compared to traditional methods [31,32].

Research indicates that different formulations of PRF can yield varying regenerative potentials, necessitating a deeper understanding of their specific applications in dentistry. For example, studies have shown that i-PRF can enhance cell proliferation and migration, making it suitable for injectable applications in regenerative dentistry [33,34]. Meanwhile, A-PRF has been noted for its ability to release growth factors over an extended period, thereby promoting sustained healing [35]. These PRF formulations have been the subject of investigation, with findings suggesting a positive effect on bone regeneration; their specific compositions can influence the rate and quality of healing [36]. As the field continues to evolve, integrating PRF into clinical practice is expected to enhance the outcomes of dental surgeries, making it a critical area of ongoing research and application in regenerative dentistry [8].

Thus, the present study was designed to compare and verify the similarity of the micro- and macroscopic aspects of APC “plugs” and membranes produced using different centrifuges and their respective centrifugation protocols; moreover, the macroscopic results and blood serum levels obtained through the analysis of the blood count and coagulogram of selected patients were correlated. The results of the present study demonstrate similarity in macroscopic terms when comparing different centrifuges to obtain PRF membranes. Therefore, no significant differences were observed in the weight and linear measurements of “plugs” and membranes after the use of different centrifugation protocols.

Our data partially corroborate Ehrenfest et al. [27]’s data, which macroscopically compared the weights and measurements of plugs and PRF membranes from four different centrifuges (the Intra-Spin Intra-Lock, the A-PRF 12-Advanced PRF Process, the Salvin 1310-Salvin Dental, Charlotte, NC, USA, and the LW-UPD8-LW Scientific, Lawrenceville, GA, USA). They selected eight volunteers and nine tubes for blood collection. The weight values of the “plugs” and the PRF membranes obtained were similar to those in the present study. The authors observed no statistical differences in macroscopic data between the Intra-Spin and Salvi Dental centrifuges. However, differences were found between the centrifuges of other commercial brands. Unlike our methodology, in this study, the authors used the same centrifugation protocol for all centrifuges (12 min at 2700 rpm). It is known that the best protocol for obtaining this is 400 G of force during processing. Hence, the authors justify the statistical differences between these factors and the difference in vibration between the centrifuges. In theory, the absence of statistical differences found in the present study can be justified by the individualization of the centrifugation protocols used in our study, where the processing was individualized to obtain 400 G of force while obtaining the PRF.

The present study’s data showed a lower variability value for centrifuges C1 and C2 compared to centrifuge C3. One explanation for this finding may be the centrifugation force and the vibration obtained during processing. Studies have observed physical and molecular differences when different vibrations occur during processing [13,27]. Enhenfest et al. [27] reported that when the rotor vibration is above one, this directly influences the size and homogeneity of the PRF membranes.

In the present study, there was a correlation between the findings of blood cellular components obtained through blood counts and the macroscopic data from the “plugs” and PRF membranes from different centrifuges. No correlations were found. When we look at the data obtained from the blood count test, it is notable that all cellular data follow the standard reference rates. Therefore, this fact may explain the lack of correlation between the data since once the patient’s blood count test is within normal limits, the PRF membrane’s weight and dimension values will be within the normal range. Average values were obtained in the present study; however, to the best of our knowledge, this is the first study that sought to correlate these factors.

Regarding the venous blood collection time, the data were similar to those obtained by other studies [27,37]. In the present study, the average collection time, regardless of the centrifuge, was 21.5 ± 9.9 s; a value close to that obtained by other authors, which was 22 s [27]. Thus, this standardization can also justify the similarity in weights and measurements obtained in the present study.

### Study Limitations

The limitations of the present study are the small number of patients included and the lack of molecular analysis for the PRF membranes obtained by the different centrifuges studied; even though this was not the study’s goal, it could help in a deep assessment and comparison. Several authors have reported different biological signatures obtained from different centrifuges for PRF [27,28,37,38]. These authors reported different concentrations of growth factors such as platelet-derived growth factors (PDGFs), transforming growth factor β (TGF-β), vascular endothelial growth factor (VEGF), epidermal growth factor (EGF), and insulin growth factor-1 (IGF-1); however, the different growth factor molecular analyses could help in the understand of the fundamental differences or similarities between membranes obtained in the present study. Therefore, it is essential to highlight that no data in the literature define the best values and ideal concentrations of these growth factors present in PRF membranes. Furthermore, the influence of these different levels of growth factors must be better tested clinically.

## 5. Conclusions

Within the limitations of this study, it is possible to affirm and conclude that different centrifuges achieved similar results for PRF preparation (in terms of length and weight), and no correlation was achieved between the PRFs and blood serum data presented by the patients.

## Figures and Tables

**Table 1:** Coefficient of variability for the macroscopic results according to the centrifuge used.

Centrifuge	Wet PRF (Initial) Weight	Wet PRF (Initial) Length	PRF Membrane Weight	PRF Membrane Length
C1	20%	11%	20%	12%
C2	24%	15%	21%	22%
C3	33%	22%	36%	33%

**Table 2:** The average of the results obtained for the blood serum components.

Blood Serum Value	Average Result	SD	Standard References
Erythrocytes (millions/μL)	5.025	0.287	4.5–5.5
Hemoglobin (g/dL)	14.72	0.604	13.0–17.0
Hematocrit (%)	43.87	1.573	40.0–50.0
Platelets (thousand/mm^3^)	238,666.66	45.467	150–400 mil/mm^3^
Total leukocytes (thousand/mm^3^)	7.14	0.889	4.5–11.0
Neutrophils (thousand/mm^3^)	4.28	0.452	1.80–7.70
Total lymphocytes (thousand/mm^3^)	2.48	0.291	1.0–4.0
Monocytes (thousand/mm^3^)	0.37	0.047	0–0.8
Prothrombin time (seconds)	10.99	0.484	11.0–13.5
Prothrombin activity (%)	98.90	1.621	70–130%
Activated partial thromboplastin time (seconds)	25.25	0.626	25.0–34.0
Bleeding time (minutes)	2.1	0.206	1.0–4.0 min

## Data Availability

The original contributions presented in this study are included in the article. Further inquiries can be directed to the corresponding author.

## Abbreviations

3D	Tridimensional
APCs	Autologous Platelet Concentrates
CTG	Connective Tissue Graft
HA	Hydroxyapatite
L-PRF	Leucocyte- and Platelet-Rich Fibrin
L-PRP	Platelet- and Leukocyte-Rich Plasma
mg	Milligram (s)
mm	Millimeter (s)
P-PRF	Pure Platelet-Rich Fibrin
P-PRP	Pure Platelet-Rich Plasma
PDGF	Platelet-Derived Growth Factor
PRF	Platelet-Rich Fibrin
SD	Standard Deviation
TGF-β	Transforming Growth Factor-Beta
β	Beta
β-TCP	β-tricalcium phosphate
